# The HCR-20 and violence risk assessment – will a peak of inflated expectations turn to a trough of disillusionment?

**DOI:** 10.1192/bjb.2020.14

**Published:** 2020-12

**Authors:** Edward Silva

**Affiliations:** Ashworth Hospital, Mersey Care NHS Foundation Trust, Liverpool, UK

**Keywords:** Risk assessment, violence, forensic, HCR-20, SPJ

## Abstract

The HCR-20 has taken on a life of its own. In forensic services it has been elevated from helpful aide-mémoire into a prophetic tool worthy of Nostradamus himself. Almost every outcome is interpreted through it. Despite the evidence of its limited utility, the difficulties of predicting rare events, the narrative fallacies and other heuristic biases it creates, and the massive opportunity costs it entails, commissioners and services alike mandate its use. Yet in routine practice the problems are not acknowledged, multiple conflicts of interest lie unobserved and other opportunities are neglected.

Violence risk assessment is a core part of forensic psychiatry. It has evolved from an unstructured clinical and anecdotal exercise, through the use of actuarial tools and is now dominated by a variety of structured professional judgement (SPJ) instruments. Of these the Historical Clinical Risk Management-20 (HCR-20) is pre-eminent. It has itself evolved: first published in 1995, it is now in its third iteration. Initially it was used as an aide-mémoire to assist clinicians and others to systematically assess what were believed to be risk factors for violence across time: historical (ten items), clinical and risk management (five each). In 2001 further materials were added, including scenario planning.

The HCR-20 is the most widely used violence risk assessment tool in the world,^1^ and in the UK it has become the ubiquitous gold standard for the risk assessment of violence in forensic services. NHS England commissioners of secure services for forensic patients mandate an HCR-20 assessment, updated every 6 months, even when there is no history of violence. As can be the case with many expert judgements,^2^ any outcome can be seen through its lens. In cases of disaster, ‘the HCR-20 was completed incorrectly’, ‘the recommendations were not followed’, ‘it was not updated on time’ and, most seriously, ‘there was no HCR-20’. When there is success then the merits of the risk assessment and assessor are praised. Fearing blame in the event of failure, my psychologist colleagues spend dozens of hours reading through volumes of notes and the outputs are so long as to be unreadable. Explanations of previous violence are formulated, estimates of risk made and future risk scenarios hypothesised. The tool is over-relied on to guide patient management through complex systems of care, a task it cannot achieve. Curiously, updates are frequently done after clinical decisions about management have been made. But the limitations are not acknowledged and they are legion. Some are relevant to violence risk in general and others specifically to SPJ tools and the HCR-20 in particular.

## Limitations to SPJ tools

• There is no grade 1 randomised controlled trial (RCT) evidence for the effectiveness of SPJ tools in reducing violence; the only RCT tested the Short Term Assessment of Risk and Treatability (START) and gave a negative result.^3^
• Most items in structured risk assessment instruments, especially the Psychopathy Checklist –Revised (PCL-R), and many in the HCR-20 do not predict violence.^4^
• Random combinations of risk factors are as useful as those assembled in standardised instruments.^5^
• The HCR-20 ignores pertinent facts regarding the importance of adherence to specific drug treatments and risk.^6^
• The area under the curve (AUC) measure of utility bears very little relevance to use in clinical practice and ignores the difficulty of prediction when base rates are low.^7^ It is a concept rarely used in other areas of medical practice, where positive predictive value (PPV) is the usual measure.
• As with any attempt to predict rare events^8^ (p. 170) the PPV of the HCR-20, as with other risk tools, is poor and it produces many more false- than true-positive findings.^9^
• High-quality negative evidence regarding the utility of multiple risk tools is not noticed, is refuted and as yet has had no impact on commissioners or services.^9,10^
• Intellectual and financial conflicts of interest in the publications on various SPJ tools are not mentioned.^11^ Those who submit research papers on the HCR-20 and other risk instruments rarely, if ever, declare an interest in receiving fees from training in its use. Yet it is a ‘product’, like a pharmaceutical agent, and one for which they stand to gain financially if it is promoted. Similar conflicts may exist for those who conduct serious adverse incident reviews recommending improved use of risk assessment if this is also a service they provide on a commercial basis.
• The narrative explanations of risk formulations and future risk scenarios are accepted. They are not seen as rhetorical devices requiring empirical validation, unlikely to be correct in systems too complex for analysis. To make sense of the world humans require stories that examine concrete events, ignoring chance and the things that did not happen. Any recent salient event is a candidate to become the kernel of a narrative explanation.^12^
• Narratives combined with recent or high-profile events feed heuristic biases, including representativeness, availability and, most important, affect.^13,14^ In forensic services our patients have often violated basic human norms: rape, incest, murder, mutilation and losses of control.^15^ At times we will be disgusted. This is rarely acknowledged and instead there is a serious risk that an emotionally driven sense of disgust^14^ will result in the immediate generation of opinions for which the supporting evidence is subsequently found, with risk assessment becoming confused with the assessment of outrage^16^ and becoming a moral exercise.^17^
• Whatever our organisations may tell us, it feels as if there is only punishment for failure and so an increasing tendency to risk aversion is inevitable.^18^
• The definition of violence used in the HCR-20 is so broad (including verbal threats) as to be meaningless in the services we work in.

## The consequences of ignoring these limitations

Ignoring these difficulties is not just a failure of a tool. It has enormous consequences for patients, professionals, the public and those who pay for our services. The patients we care for face prolonged detention and the opportunity cost of professional time that could be spent delivering interventions. The patients we do not care for face delays in accessing care, often untreated and in inadequate facilities in prison. As professionals we become preoccupied with avoiding failure instead of achieving improvement and it often feels like the risk that is being managed is the risk to ourselves and to, or even from, our organisations. An explicit analysis of risk will be an important part of a patient's treatment, but in the context of deficiencies in treatment and access to care, an HCR-20 will not protect us, or our organisations, from litigation or public criticism. Instead of trying to determine what the prospective risk is given the facts and the base rates, we anticipate how failure will be perceived in hindsight. Those that fund our services complain that too many are detained,^19^ while removing funding from objective research.^20^ Inquires^21^ continue to recommend interventions that do not work – case management,^22^ risk assessment and community treatment orders^23^ – and themselves can fuel narrative fallacies.^24^ Through our overvalued ideas regarding risk assessment, forensic services are left caring for a tiny percentage of mentally disordered offenders, who we dare not part company with, and at vast expense.^25^

## What can we do?

The argument is not that risk assessments should be abandoned, only that we should be much more circumspect about their power, utility and explanatory value, and recognise how narratives may mislead as well as explain. This is now the position in the related field of suicide risk assessment. In stark contrast to the requirements for secure services and the use of the HCR-20, the National Institute for Health and Care Excellence (NICE) advice is: ‘Do not use risk assessment tools and scales to predict future suicide or repetition of self-harm’^26^ (p. 8), for the simple reason that we cannot stratify risk using the tools available. The information they provide regarding the likelihood of the outcomes we are really concerned about is of no practical use.^27^ But it is very hard for systems to change and for professionals to give up their sincerely held beliefs. This is the case throughout medicine. It takes an average of 17 years to translate research findings into practice.^28^ Although short structured assessments would be helpful, our attempts to stratify risk of violence are not useful and should be abandoned, as should narrative explanations of the past and hypothesising future scenarios. It is not particularly useful to say that a man who has been violent in the past might be violent in future if intoxicated, threatened, feeling disrespected or aggrieved, lost to follow-up, non-adherent to antipsychotic or mood stabilising medication and in contact with a vulnerable potential victim.

Some hope that technology will provide a solution. But it took the resources of Deep Mind's artificial intelligence (AI) capabilities, combined with a vast sample of over 700 000 patients, to develop a system to predict the highly specific outcome of acute kidney injury within the tight window of 48 h in highly monitored in-patient environments.^29^ So why do we think that we can predict violent behaviour over timescales of weeks, let alone months or years, on the basis of human analysis, or that in future AI will be able to make longer-term predictions about far more complex human behaviours? Even if such analytic systems are developed, it is questionable whether clinicians, patients or the legal system would accept them. It is likely that highly discriminatory variables would be key factors in AI algorithms – gender, age, ethnicity, residence in a high crime area, peer group criminality – and there would be fears that the scenarios of *The Minority Report* would emerge.^30^ Instead the approach adopted by NICE regarding suicide and self-harm should be taken, with the emphasis on the delivery of effective treatments, ensuring services are adequately resourced and developing better habits regarding quality.^31^

## A hint of change?

A quick search using Google Trends shows that online interest in the HCR-20 has fallen dramatically, from a peak in September 2007 to date. The Gartner Hype Cycle,^32^ with its phases of a technology trigger, a peak of inflated expectations, a trough of disillusionment, a slope of enlightenment and then a final plateau of productivity, is held as an example of the boom, bust and then stabilisation of new technologies. But perhaps this is what is happening already?

## About the author

**Edward Silva** is a consultant forensic psychiatrist at Ashworth Hospital, Mersey Care NHS Foundation Trust, Liverpool, UK. He has worked in secure services since 1998 and has been involved in the use of SPJ tools throughout as part of the routine clinical care of many detained patients.
